# Supplementary material for: Awareness and Availability of Low Sodium Iodized Salt: Results from Formative Research of Promoting Uptake of Low SodiUm Iodized Salt by Rural and Urban HousehoLds in India—The PLURAL Study
Source: Nutrients. 2023 Dec 30;16(1):130. doi: 10.3390/nu16010130 (PMC10781031; doi:10.3390/nu16010130)
Supplement: Supplementary file 1 [file nutrients-16-00130-s001.zip › nutrients-2759793-supplementary.pdf]

Promoting uptake of Low sodiUm iodized salt by Rural And urban househoLds in India: The PLURAL study

Topic guide for interview with consumers

Date: Start time: Place and venue:

Instruction – Before the start of the interview, please seek written informed consent for participating in the interview and audio recording of the interview.

Before the start of interview, please introduce yourself. Please brief the interviewee about the purpose of the study and the purpose of the interview. Kindly reiterate that participation is purely voluntary and the information that they provide will be kept confidential with access to only the study investigators. Please also inform that their names will not be revealed while reporting the results. Please let them know that the interview may last for 30 to 45 minutes. Please ask the interviewee if they have any queries and clarify them before proceeding with the interview.

Please note that the interview guide is just an aid to conduct the interview. Based on the participant and the response, you can change the questions and probes. Please do note the changes you have made.

Before starting the audio recording, state "Audio recording started."

Interviewee age: Interviewee gender: Interviewee number:

Good morning/afternoon,
I am ______________, working at the Public Health Foundation of India. We are conducting a study regarding the use of low sodium iodized salt and ways to improve its consumption. In this regard, I wanted to talk to you about salt intake, diseases due to high salt intake and interventions for salt reduction. We want to get your opinions on low sodium iodized salt as a potential way for salt reduction, what could be the challenges and benefits, and how we increase its usage in the community.

Hypertension and Salt Intake

1. What do you know about hypertension or high blood pressure and its causes? (Probe for risk factors especially dietary risk factors i.e., salt and fruits and vegetables, symptoms, signs, treatment)

2. Please describe the lifestyle changes that can be made to reduce BP. (Probe for dietary changes like fruits and vegetable intake, physical activity, and salt intake)

3. What is the salt usage in your household like? Who usually buys it? What are the reasons for purchasing the type/brand of salt? (Probe for salt consumption, type of salt bought, brand and cost, advertisements of salt brands)

4. Have you or your family member been asked to reduce salt? What has been your experience when trying to reduce salt on your own or on advice? (Probe for benefits of dietary salt reduction)

5. What do you think generally about reducing salt in diet? What difficulties do you think people face while trying to replace or reduce salt? (Probe for difficulty in reducing salt in diet, for salt replacement, salt reduction, consumption of high salt food items like pickles, chutneys, processed foods)

About LSIS

6. Nowadays, in shops low sodium salt packets are being sold. Have you seen it in your local shop? What do you think about this type of salt? Have you ever tried it? (Probe for availability, cost, benefits over conventional salt, perceptions about the salt like quality, taste, color etc., any harmful effects of low sodium compared to conventional salt)

7. We will soon start an intervention to promote low sodium salt amongst people residing in your area. What strategies do you think we should adopt to promote uptake of low sodium salt? (Probe for different types of communication, role of subsidies, interventions at the point of purchase, previous experience with health promotion activities)

8. Is there anything else that you would like to add?

Summarize the interview and read back important points of the interview. Thank the interviewee for participating in the interview.

Interview end time: _______________________

<u>P</u>romoting uptake of <u>L</u>ow sodi<u>U</u>m iodized salt by <u>R</u>ural <u>A</u>nd urban househo<u>L</u>ds in India: The PLURAL study

<u>Topic guide for interview with retailers</u>

Date: Start time: Place and venue:

Instruction – Before the start of the interview, please seek written informed consent for participating in the interview and audio recording of the interview.

Before the start of interview, please introduce yourself. Please brief the interviewee about the purpose of the study and the purpose of the interview. Kindly reiterate that participation is purely voluntary and the information that they provide will be kept confidential with access to only the study investigators. Please also inform that their names will not be revealed while reporting the results. Please let them know that the interview may last for 30 to 45 minutes. Please ask the interviewee if they have any queries and clarify them before proceeding with the interview.

Please note that the interview guide is just an aid to conduct the interview. Based on the participant and the response, you can change the questions and probes. Please do note the changes you have made.

<u>Before starting the audio recording, state "Audio recording started."</u>

Interviewee age: Interviewee gender: Interviewee number:

Good morning/afternoon,
I am ______________, working at the Public Health Foundation of India. We are conducting a study regarding the use of low sodium iodized salt and ways to improve its consumption. In this regard, I wanted to talk to you about salt intake, diseases due to high salt intake and interventions for salt reduction. We want to get your opinions on low sodium iodized salt as a potential way for salt reduction, what could be done to make it more available through shops, and how we increase its usage in the community.

Hypertension and Salt Purchase Behavior

1. What do you know about hypertension or high blood pressure and its causes? (Probe for risk factors especially dietary risk factors i.e., salt and fruits and vegetables, symptoms, signs, treatment)

2. Please describe the process of acquiring, buying, and selling of salt in your shop including the margins on it. (Probe for supply chain of salt, brands that they sell, popularity of different brands, cost of salt and margins on sale, ordering and storage process)

3. Please describe the purchase behavior and preferences of consumers when it comes to salt. (Probe for brand, type of salt, quantity purchased, cost)

About LSIS

4. Recently companies have rolled out low sodium salt into the market. What has been your experience in selling this type of salt? If you have not been selling, what do you know about this type of salt? Is it easily available? (Probe for its benefits over conventional salt, supply chain of low sodium salt, brands, cost, availability, sales compared to conventional salt, profit margin, consumer awareness and demand)

5. We will soon start an intervention to promote low sodium salt in this area. What strategies do you think we should adopt to promote uptake of low sodium salt? (Probe for different types of communication, role of subsidies, interventions at the point of purchase, previous experience with health promotion activities)

6. Is there anything else that you would like to add?

Summarize the interview and read back important points of the interview. Thank the interviewee for participating in the interview.

Interview end time: ________________________

<u>P</u>romoting uptake of <u>L</u>ow sodi<u>U</u>m iodized salt by <u>R</u>ural <u>A</u>nd urban househo<u>L</u>ds in India: The PLURAL study

<u>Topic guide for interview with influencers</u>

Date: Start time: Place and venue:

Instruction – Before the start of the interview, please seek written informed consent for participating in the interview and audio recording of the interview.

Before the start of interview, please introduce yourself. Please brief the interviewee about the purpose of the study and the purpose of the interview. Kindly reiterate that participation is purely voluntary and the information that they provide will be kept confidential with access to only the study investigators. Please also inform that their names will not be revealed while reporting the results. Please let them know that the interview may last for 30 to 45 minutes. Please ask the interviewee if they have any queries and clarify them before proceeding with the interview.

Please note that the interview guide is just an aid to conduct the interview. Based on the participant and the response, you can change the questions and probes. Please do note the changes you have made.

<u>Before starting the audio recording, state "Audio recording started."</u>

Interviewee age: Interviewee gender: Interviewee number:

Good morning/afternoon,

I am ______________, working at the Public Health Foundation of India. We are conducting a study regarding the use of low sodium iodized salt and ways to improve its consumption. In this regard, I wanted to talk to you about salt intake, interventions for salt reduction. We want to get your opinions on low sodium iodized salt as a potential way for salt reduction, challenges in prescribing and using low sodium salt among people in general and patients with cardiovascular disease and chronic kidney disease in particular.

1. Since how long have you been practicing here/working here? What is your experience with patients diagnosed with hypertension? (Probe for number of patients with hypertension, their diagnosis and management, adherence to treatment, lifestyle changes like physical activity, stopping alcohol and tobacco use, healthy diet, fruit and vegetable intake)

2. What are your thoughts on salt reduction for hypertension management? (Probe for importance of salt reduction, advice to patients on salt reduction, how to reduce salt in the diet)

3. What can you say about patient's willingness and action regarding reducing salt in the diet? (Probe for motivation and challenges to reduce salt intake, use of other types of salts like Himalayan salt and sendha namak)

4. What is your opinion regarding low sodium salt as a strategy to reduce salt consumption? (Probe for feasibility, challenges, potential dangers of consuming low sodium salt certain group of patients, familiarity with guidelines on low sodium salt)

5. Do you have any reservations/concerns on advising low sodium salt to patients?

6. We will soon start an intervention to promote low sodium salt amongst people residing this area. What strategies do you think we should adopt to promote low sodium salt? (Probe for different types of communication, role of subsidies, interventions at the point of purchase, previous experience with health promotion activities)

7. If we were to involve you in the intervention to promote low sodium salt will you be interested in participating? If yes, how do see foresee your participation? (Probe for patient education, giving pamphlets, pasting posters outside the clinic/shops, clarifying doubts of patients, participate in meetings)

8. Is there anything else that you want to add/discuss?

Summarize the interview and read back important points of the interview. Thank the interviewee for participating in the interview.

Interview end time: ______________________
